# Neuroanatomy, episodic memory and inhibitory control of Persian-Kurdish simultaneous bilinguals

**DOI:** 10.1038/s41598-024-79955-2

**Published:** 2024-11-25

**Authors:** Samira Golshani, Olga Kepinska, Hamid Gholami, Narly Golestani

**Affiliations:** 1 Department of ELT, Kermanshah Branch, Islamic Azad University, Kermanshah, Iran; 2 Brain and Language Lab, Vienna Cognitive Science Hub, University of Vienna, Vienna, Austria; 3 Department of Behavioral and Cognitive Biology, Faculty of Life Sciences, University of Vienna, Vienna, Austria; 4 Brain and Language Lab, Department of Psychology, Faculty of Psychology and Educational Sciences, University of Geneva, Geneva, Switzerland

**Keywords:** Bilingualism, Brain structure, Grey matter volume, Episodic memory, Cognitive neuroscience, Hippocampus, Neuroscience, Long-term memory

## Abstract

We assessed simultaneous bilinguals and monolinguals on inhibitory control and episodic memory, and assessed their grey matter volumes in brain regions known to be involved in language processing, executive control and memory. Bilinguals outperformed monolinguals on episodic memory, and performance on the memory and inhibition tasks were correlated, only in the bilingual group. This suggests that the bilingualism-related benefits on memory are related to individual differences in executive control. We found larger grey matter volumes in bilinguals in left pars opercularis and in bilateral SFG, caudate nuclei, and parasubiculum. Episodic memory performance was correlated with volumes of bilateral posterior hippocampi, again only in the bilinguals, again suggesting that bilingualism may be driving this effect. Finally, we found positive structural covariance between the volumes of the bilateral parasubiculum and that of important components of the executive control network. We provide a novel, mechanistic explanation accounting for observed behavioural advantage and brain structural differences: bilingualism may boost the prefrontal cortex-hippocampal neural circuitry commonly underlying both executive control and memory, via cascade and reverberant effects, leading to synergistic benefits in both cognitive domains. This new framework has important implications for protective effects on cognition and brain health in relation to second language learning.

**Supplementary Information:**

The online version contains supplementary material available at 10.1038/s41598-024-79955-2.

## Introduction

The posited executive control benefits of bilingualism on measures of inhibitory control, working memory, task switching and conflict resolution (see^1^ for reviews) remains hotly debated, as previous studies on this topic have yielded mixed results^2,3^. Besides executive functioning, studies also point to memory benefits of bilingualism, with reports of better episodic memory in bilingual compared to monolingual children^4^ and older adults^5,6^. Bilinguals may show enhanced episodic memory compared to monolinguals because when one masters two languages, there is a greater need to integrate and/or organize the information from both languages in memory^4^. This suggestion originally came from earlier work, which had proposed that in bilingual children, memories that are encoded in the context of one language are intentionally or automatically accessible in the other language, conferring greater flexibility in the use of information linked to specific remembered events across the two languages in memory^7^. Conversely, the need to keep the two languages functionally distinct in bilingualism results in changes to the cognitive architecture underlying language and memory, with improved retrieval of episodic memories when using the language that was used during the encoding of those same experiences^8^. Better declarative memory in bilinguals may also be due to the fact that they have to represent, learn, and process two languages per se as compared to monolinguals^9^. Indeed, language learning involves vocabulary acquisition, which depends on rote memory skills^10^, and previous studies have shown that second language learning in children and adults can improve the encoding and recall of words or pictures^5,11^.

Consistent with behavioural evidence for both executive control and memory advantages in bilinguals, structural brain imaging work has shown larger regional volumes in this group not only in cortical and subcortical networks involved in language processing but also in ones involved in executive control and in declarative and episodic memory^9,12,13^. It is well established that the hippocampus plays a crucial role in different forms of explicit memory encoding. It is involved in episodic memory in domains ranging from perception, language, and empathy to problem solving, but also in (among others): rote learning^14^, word learning, spatial memory and navigation^15^, and in language processing more generally^16^. Interestingly, longitudinal increases in the volume of the hippocampus as a function of foreign language learning have also been reported^17,18^, along with changes in its shape^13^. Cross-sectional studies have also shown slower age-related decline in left or bilateral hippocampal volumes in bilinguals compared to monolinguals^6,19^. Furthermore, functional brain imaging studies show that initial stages of language learning (of vocabulary and grammar alike) highly depend on the involvement of the hippocampus both in terms of its relative levels of activation^20,21^ and in terms of functional connectivity^22^. Together with investigations having shown that in Alzheimer’s patients, the onset of symptoms is delayed in bilinguals compared to monolinguals^23^, these findings support the idea that bilingualism is an experiential factor that confers cognitive and neural reserve, leading to behavioural and neural differences that are more apparent during aging. One previous study has investigated the effects of bilingualism on memory and executive control in older bilingual adults^5^, but the neural underpinnings underlying this relationship have not been elucidated’.

Executive control, constituted of inhibition, working memory, cognitive flexibility and conflict resolution, is known to rely on a network including the medial prefrontal cortex (mPFC), the dorsolateral prefrontal cortex (DLPFC) which straddles the middle (MFG) and superior frontal gyri (SFG), the supplementary motor area (SMA) and the pre-SMA, the anterior cingulate cortex (ACC) along with regions such as the caudate nucleus and the cerebellum^24^. Studies on inhibitory control and more specifically on response conflict have also shown involvement of the hippocampus^25^. Interestingly, it is known that there are reciprocal projections between neurons in the mPFC and subcortical regions such as the hippocampus and that this hippocampal-prefrontal cortex circuit plays a very important role in cognitive and emotional regulation, and also in memory consolidation and in the integration of information about objects, place and time in the construction of episodic memory^26,27^. Further, recent work involving path analysis on brain structural data in people spanning a wide age range (24–85) has revealed that aging affects the volume of the left hippocampus (i.e. decline in volume), which then has a cascade effect on the volume of the left SFG, leading to poorer cognitive control measured via the Wisconsin Card Sorting Task, which assesses higher order flexibility and maintenance abilities^28^.

Bringing together the work showing that bilingualism confers cognitive and neural resilience against age-related decline in brain regions including the hippocampus, and the above-described posited cascading neural mechanism of decline in memory and executive control during aging, we propose a novel, mechanistic explanation that would account for both the behavioural advantages of bilingualism on aspects of executive control and episodic declarative memory. The executive control benefits of bilingualism, when found, have been attributed to continual demands on the selection and inhibition of target and non-target languages in bilinguals^1^. Another possible, *non-exclusive* explanation for the behavioural benefits of bilingualism – both in the domains of executive function and of memory – is that bilingualism may boost the prefrontal cortex – hippocampal neural circuitry commonly underlying both executive control and memory, via cascade and reverberant bottom-up and top-down effects, leading to synergistic benefits in both cognitive domains. More specifically, it could be that the hippocampal plasticity that is triggered by language and vocabulary learning^17,18^ leads to beneficial cascade effects on higher-level prefrontal executive control brain regions, which in turn further interact with the hippocampus, working hand in hand with the language network to allow for effective and efficient speech comprehension and production but also language control in the bilingual brain. Conversely, better executive control arising from the language management requirements of bilingualism could lead to improvements in the encoding and retrieval in episodic memory (cf^5^). In other words, we propose that the bolstering arising both from the continual language selection and inhibition requirements of bilingualism but also from the greater memory requirements of mastering multiple linguistic repertories and linking them to lexico-conceptual information likely interact, and lead to concomitant benefits in memory and executive control, and in their neural underpinnings. The present study aims to address this proposed mechanistic explanation of the bilingual advantage for both executive skill and memory using behavioural but also brain structural imaging data from bilingual and monolingual participants.

Most previous studies investigating the relationship between episodic memory and/or bilingualism and the hippocampus have examined the hippocampus as a unitary region. The hippocampal formation, however, consists of distinct subregions, the functionality of which is being increasingly investigated^29^. Although the role of the hippocampus in language is established^20–22^, to our knowledge, there are to date no published functional neuroimaging studies on the role of different hippocampal subregions in language. There is evidence however for a preferential role of the anterior hippocampus in conceptual, gist and semantic and maybe even lexical information, and for a preferential role of the posterior hippocampus in detailed/episodic/contextual information^30^. For example, the volume of the head of the hippocampus is positively associated with verbal memory performance in healthy older adults^31^. Also, functional neuroimaging has shown greater activation of anterior hippocampus during conceptual category fluency and of posterior hippocampus during retrieval of spatio-perceptual information^32^. This is consistent with work having shown that the anterior hippocampus has direct and reciprocal connections with regions including the anterior temporal lobes, known to be critical for conceptual representations and semantic memory^30^. Further evidence comes from a recent fMRI study revealed that mid-anterior hippocampus and the entorhinal cortex represent the inferred hierarchical structure between words, with greater activation similarity between subordinate-level words and their related superordinate than to unrelated ones^33^. Finally, a new meta-analysis of direct brain recordings has implicated the anterior medial and more specifically the rhinal cortex in the N400 ERP response, known to be elicited by words and potentially meaningful stimuli; this has led to a new, unifying model suggesting that the anterior hippocampus and the perirhinal cortex are important parts of the ‘what’ ventral lexico-semantic processing stream in declarative memory^34^.

There are also a growing number of studies on the differential role of specific (para-)hippocampal subfields in episodic memory using tasks involving spatial (and temporal) information. Although one study in healthy young adults showed comparable levels of activation across multiple subfields of the hippocampus proper during retrieval^35^, other studies having looked at the whole hippocampal formation point to a specific involvement of anterior medial portions of the hippocampus, and of the anterior presubiculum and of the parasubiculum of the parahippocampus, in particular when scene-based information is involved^36,37^. The latter aligns with work having implicated the parasubiculum in episodic memory retrieval from autobiographical memory^38^, and in the mental construction of internal scenes during episodic memory recall^39^. Other studies have however shown a role for posterior hippocampus subregions in the retrieval of autobiographical memory, specifically in people with poorer memory recall^40^. Further, it has been proposed that although all hippocampal subfields may be similarly involved in memory encoding, the posterior hippocampus may be more involved in memory retrieval^41^. In sum, existing links in the literature between hippocampal subfields in language knowledge, episodic memory and spatial aspects of episodic memory point to the preferential involvement of the anterior hippocampus in language knowledge, and of the anterior hippocampus, parasubiculum and posterior hippocampus in different aspects of episodic memory (i.e., the former two during spatial tasks, and the latter more during retrieval).

In this present study, we analysed behavioural and brain structural imaging data from Persian-Kurdish simultaneous, non-elective bilinguals and Persian monolinguals. Kurdish and Persian are linguistically sister languages, both belonging to the Western branch of the Indo-Iranian language family. They differ more in vocabulary and syntax than in phonology. Lexical dissimilarity was reported at 48.5% (Mohammed et al., 2023). We assessed whether simultaneous bilingualism is associated with better inhibitory control, measured via performance on the Victoria Stroop Test (VST), and with better episodic memory, measured using the What-Where-When (WWW) test^42^. We also tested whether individual differences in the two tasks were correlated; such a finding would support the idea that bilingualism-related improvements on executive function and memory go hand in hand, i.e. that there is an inherent relationship between the two domains, possibly due to concurrent plastic changes in brain regions underlying the two cognitive domains. Given that our participants were young healthy adults, it was not clear whether we would observe better performance in the bilinguals, or whether due to ceiling effects we would not observe group differences. We also probed for differences in brain structure between groups in brain regions associated with language processing but also with executive processing and episodic memory, with the prediction of larger volumes in these regions, including the hippocampus or in subregions thereof in the bilinguals. We further tested for relationships between performance on episodic memory and the volume of the hippocampus or its subregions (see below). Given the likely preferential roles of different hippocampal regions/fields in language and episodic memory, we took a graded approach in our analyses. Specifically, for both analyses of group differences and for those relating hippocampal (subregion) volumes to memory performance, we examined the volumes of regions spanning from maximally inclusive to progressively more targeted, as follows: (a) hippocampus, (b) anterior (versus posterior) hippocampus, and (c) parasubiculum. We predicted that bilinguals would have larger volumes of anterior hippocampus. Given the possible role of different hippocampal regions in different aspects of episodic memory, we did not have strong predictions regarding which subregions would correlate most with episodic memory performance. We thus treat this question as an exploratory one. Finally, to test the hypothesis of *cascading* effects of bilingualism on brain structure potentially originating in the hippocampus and affecting executive control regions, we performed whole-brain (cortical) structural covariance analyses between the volumes of the regions that showed group differences and the rest of the brain, across all participants, with the prediction that there would be preferential structural covariance with regions involved in executive control.

## Materials and methods

### Participants

Fifty-four (*N* = 54) healthy young adults (between 19 and 31 years of age) took part in this study, but data from 2 had to be excluded (see the section on brain imaging data pre-processing). Selected mainly by public notice and word of mouth, 25 participants were Persian monolinguals (4 males), and 27 were highly proficient Persian-Kurdish simultaneous bilinguals (8 males) who had acquired both languages since they were born. The two groups were undergraduate students majoring in different fields of the humanities. They all lived in Kermanshah, a dual-language city in the west of Iran. They were from middle socio-economic backgrounds; this was determined via a detailed questionnaire that assessed each individual’s occupation, and the highest level of formal education by assigning numeric values from 1 to 10 to these domains. The groups were matched for age, sex, education and socioeconomic status (SES). A Chi-square test revealed no significant difference in the male/female ratio (χ^*2*^ = 1.35, *df* = 1, *p* = .24) between groups, and independent samples t-tests showed no difference in age (t(50) = 0.41, *p* = .68) nor in SES (*t* = -1.4, *p* = .15) between the groups. According to the Edinburgh Handedness Inventory^43^, all the participants were right-handed. To gather detailed information on their linguistic background, participants were requested to fill out a language history questionnaire^44^, which was translated into Persian. Since Arabic and English are taught at schools in Iran, we also asked them to self-rate their Arabic and English listening, reading, writing, and speaking skills. None of them were proficient in either of the two languages; see Table 1 for further details on the participants’ self-reported language background. The bilingual group reported frequent exposure to and use of both Persian and Kurdish in their daily lives. None of them played any musical instrument. The study protocol was approved by the ethics committee of Farabi Hospital, Kermanshah, Iran, written informed consent was obtained from all participants, and all procedures were carried out in accordance with guidelines and regulations of the ethics committee of Farabi Hospital, Kermanshah, Iran. All participants were remunerated for their participation. Note that even though the groups did not differ in terms of the male/female ratio, there were more women than men in both groups. Given known sex differences in aspects of language-related behaviour and the prevalence of certain language disorders (such as dyslexia), we chose to control for sex in all our analyses to account for possible differences in how our relationships of interest may manifest between men and women.

**Table 1:** Subjects’ demographics (mean (SD)).

	*Bilinguals*	*Monolinguals*
Age	23.8 (3.2)	24.2 (2.6)
Gender Male/female	8/19	4/21
Formal education (years)	14.8 (1.7)	15.05 (1.3)
SES	61.8 (5.2)	62.9 (5.2)
Age of Acquisition		
Persian	at birth	at birth
Kurdish	at birth	at birth
Persian proficiency		
Speaking*	7	7
Listening*	7	7
Kurdish proficiency		
Speaking*	6.3 (0.7)	1.2 (1)
Listening*	6.8 (0.3)	1.5 (0.9)
English proficiency*	1.4 (0.8)	1.6 (0.9)
Arabic proficiency*	1.3 (0.4)	1.4 (0.5)
Daily use of Persian (% )	43.3 (3.6)	96.8 (3.7)
Daily use of Kurdish (%)	61.5 (4.7)	4.02 (2.4)

* indicates rating scale from 1 to 7 (7 = high proficiency, 1 = low proficiency).

### MRI acquisition

Structural brain images were obtained using a 1.5T MRI scanner (Siemens Avanto), at the MRI centre of Farabi Hospital, Kermanshah, Iran. For each participant, a T1-weighted, MPRAGE image was acquired, using the following parameters: repetition time (TR) = 1460 ms, time to echo (TE) = 2.95, flip angle = 8°, field of view (FOV) = 210 mm, T1 = 900 ms, acquisition time = 6.01, matrix = 256 × 240, slice thickness = 1 mm and voxel size = 1 × 0.8 × 1 mm.

### Behavioural assessment

Two weeks after the MRI scanning session, participants individually underwent a 1 h behavioural testing session consisting of a computerized VST^45^ to assess inhibitory control, and the What-Where-When computerized episodic memory test designed by^42^.

#### The Victoria Stroop test

To measure inhibitory control, a computerized version of the VST, similar to the paper format^46^ but implemented in Psychopy^47^, was utilized. The test consists of three slides (comprised of Dots, Neutral words, and Colour words), each containing 24 stimuli in four colours: green, yellow, blue, and red. Each slide contains 6 rows of 4 items. In the ‘dots’ condition, coloured dots were presented randomly. In the ‘Neutral words’ condition, neutral Persian words (e.g., بالا (up), روز (day), اما (but), درب (door)) were shown in different colours. In the ‘Colour words’ condition, colour words (e.g., آبی (blue), قرمز (red), سبز (green), زرد (yellow)) were presented in either red, blue, green, or yellow coloured font. Note that contrary to other versions of the Stroop task, in the VST *all* the items in the ‘Colour words’ conditions were incongruent, because the focus of this test is primarily on inhibitory control under interference. Participants were instructed to name the colour of the dots or the font colour of the words on the respective slides, as accurately but also as quickly as possible. Since the Persian alphabetical system is read and written from right to left, participants had to name the colours of the dots or of the words from right to left in each row. After each slide was completed, the examiner moved to the next slide by pressing the space bar. The examiner recorded the participants’ utterances to be able to later recount the number of errors and to measure the time taken to complete each slide, as is done in the paper and pencil version of the Stroop task. The Victoria version of the Stroop test has several merits. First, it is brief, and thus the participants do not get extended practice while doing the test, so the test is therefore more likely to tap into inhibitory abilities rather than learning. Second, an interference score is calculated, allowing to control for more general differences in cognitive speed. Last, the VST allows examiners to create their stimuli; this allowed us to present Persian translations of the words in the ‘Neutral word’ and ‘Colour word’ slides. Unlike the paper and pencil version of the task, in the VST task, the instructor was required to provide an immediate correction when the participants committed an error, but only when the error was not corrected by the participants themselves. Upon completion, the VST produces three scores: time to complete part D (dots), part W (neutral words), and part C (colours). To assess inhibitory control, we computed the interference score as follows: time to complete the Colour word slide *minus* the time to complete the Dot slide^46^.

#### The What-Where-When test

Episodic memory efficiency was assessed using a computerized What-Where-When (WWW) test^42^. Previous studies on memory in bilinguals have focused on the encoding and recall of words or pictures^5,48^, and only a few have studied episodic memory^4,5^. Here, we were specifically interested in testing episodic memory, as assessed by the WWW test, which has been increasingly used in animal cognition but which in the human literature has hitherto received only scant attention. Thus, the WWW episodic memory test was utilized in the area of bilingualism for the first time.

The computerized episodic memory test is a four-session task, programmed within Psychopy^47^. The features of the WWW test are compatible with the concept of episodic memory introduced by^49^, which refers to the memory for events containing temporal and spatial information. The test consists of five phases: encoding, a ‘WWW retrieval phase’, and ‘what’, ‘where’, and ‘when’ retrieval tests. In the encoding stage, participants were asked to hide 16 food items in three complex, distinctive scenes, with the different scenes occurring within different encoding periods (called ‘days’ within the paradigm). In the retrieval phase, followed by retention intervals of 5 minutes, participants were required to place the items in the same place as they had hidden them. Afterward, in the ‘where’ retrieval phase, they observed a series of “Xs” at specific locations for 5 seconds, half of which were novel, and were asked whether they had hidden any food items in those locations or not. In the ‘what’ retrieval phase, participants were asked the question “Did you hide this item?”, and then shown different items, half of which had been hidden. The final, ‘when’ retrieval phase involved asking the participants to retrieve information regarding the order in which they had hidden the items; they were asked: “Which did you hide first?”. Participants also had to indicate the exact location of the hidden item for the response to be scored as being accurate (for more details on this scoring procedure, see^42^). The subjects received no explicit instruction before or during the test that might indicate to them that they were doing a memory test, this being a criterion for an episodic memory test.

### Behavioural data analysis

To assess the differences between bilinguals and monolinguals, we first examined the assumption of normality of the behavioural and brain data. The values of skewness and kurtosis were between + 1.96 and − 1.96, thus the data was normally distributed. ANCOVA tests, controlling for sex, were run to test for group differences in performance on the episodic memory test and the Stroop test of interference and inhibitory control. Partial correlations, controlling for sex, were calculated to test for relationships between episodic memory and inhibitory control performance across the groups combined, and also in the bilinguals and monolinguals separately. The partial correlations were 1-tailed, due to a priori predictions of a positive relationship between performance across the two domains.

### Brain imaging data analysis

#### Preprocessing

All the data was first preprocessed using the structural image processing pipeline of FreeSurfer version 6^50^. Two MRIs out of 54 images were excluded, due to incidental findings. Previous papers have described this pipeline in detail, but briefly, it included the following steps: motion correction, intensity normalization, Talairach registration, skull stripping, segmentation of subcortical white matter, tessellation of the GM/white matter boundary, automated topology correction and surface deformation. On completion of the cortical models, subjects’ cortical folding patterns are registered to a spherical atlas according to folding patterns, to match cortical geometry across individuals. Then the cerebral cortex is parcellated into units based on gyral and sulcal structure, allowing local curvature and surface area measures to be computed. FreeSurfer can automatically label the cortex to replicate the labeling of a trained anatomist^50^. We used the Destrieux atlas for extraction of the cortical regions-of-interest (ROIs), and extracted subcortical measures (i.e. the caudate nuclei, hippocampi) using the Freesurfer volume-based subcortical stream^51^. Subsequently, automated subfield segmentation of the hippocampus was conducted using the hippocampal subfield segmentation toolbox within FreeSurfer^52^.

#### Region-of-interest (ROI) analyses

Based on the literature^53^, we included the following non-hippocampal ROIs known to be involved in language processing and also in executive control: the left hemispheric pars opercularis, supramarginal gyrus and middle frontal gyrus (MFG), and the bilateral caudate nuclei, superior frontal gyri (SFG), and anterior cingulate cortices (ACC).

In testing for group differences between bilinguals and monolinguals in the volume of the bilateral hippocampus and of its subregions, and for testing relationships between these and episodic memory performance, we took a graded approach in the extent of our ROIs, going from (a) the most inclusive (whole hippocampus), to (b) intermediate – i.e., anterior hippocampus, and (c) most restrictive ROI: the parasubiculum. We predicted group differences in the anterior hippocampus (and/or in the parasubiculum), but did not have strong predictions for the correlations with episodic memory. We therefore additionally included the posterior hippocampus in our analyses, to explore its potential relationship with episodic memory performance. Anterior and posterior hippocampi were segmented as follows: we divided the hippocampal masks into anterior and posterior by first, dilating the whole template-space left and right hippocampal masks 10 times and cropping them along the middle voxels in the anterior to posterior direction in Freeview (within the Freesurfer software). These template-space anterior and posterior hippocampal masks were then registered to individual subject space using mri_vol2vol, and intersected with individual subject’s whole hippocampal masks with mris_calc, resulting in separate subject-specific anterior and posterior hippocampus masks. Finally, we extracted information on the volume of each subject’s masks using mri_segstats. Here, we again used a MANCOVA, controlling for sex and whole brain cortical volume, to examine group differences in the grey matter volumes of progressively more selective ROIs, starting with the whole hippocampus, to the anterior and posterior hippocampi, to the parasubiculum.

We used a MANCOVA, performed using Jamovi (an open-source statistics software)^54^, to test for group differences in the different ROIs, while controlling for sex and whole hemispheric volume. For analysing the relationship with episodic memory performance, we ran partial correlations, controlling for cortical volume and sex, between the hippocampal ROIs and episodic memory performance, in both groups combined and in the two groups separately. We used 1-tailed tests, due to the predicted positive relationship between hippocampal and/or subregion volumes.

#### Structural covariance analysis

To test for the possible cascading effects of this group difference on the structure of the whole brain, we investigated whether the hippocampal sub-regions whose volume differed between bi- and monolinguals (i.e., the left and right parasubiculum) showed any structural covariance with other brain regions. Using FreeSurfer’s mri_glmfit tool, we therefore performed two whole-brain, vertex-wise structural covariance analyses across all participants, with the volume of the left and right parasubiculum as the seed regions (since these were found to be larger in volume in bilinguals compared to monolinguals, see Results section), controlling for the sex of the participants Cortical volume maps were smoothed using a smoothing kernel of a 10 mm full width at half maximum. Statistical significance levels were set at a *p*-value of less than 0.05. A cluster-wise correction for multiple comparisons was performed using the Monte Carlo correction (significance level, 0.05; two-tailed). All analyses were performed on each hemisphere separately. Results were Bonferroni corrected for the testing of two hemispheres and for two ROIs.

## Results

### Behavioural results

#### Group differences in memory and inhibitory control skill

Performance on the test of episodic memory was significantly different between groups (*F* (1, 49) = 7.83, *p* = .007), with the bilinguals outperforming the monolinguals. Performance on the VST task however did not reveal significant group differences in the interference score (*F* (1,49) = 0.59, *p* = .46). Thus, although we found bilingual benefits for episodic memory, we did not find benefits for inhibitory control.

#### Relationship between memory and inhibitory control

**Fig. 1:**
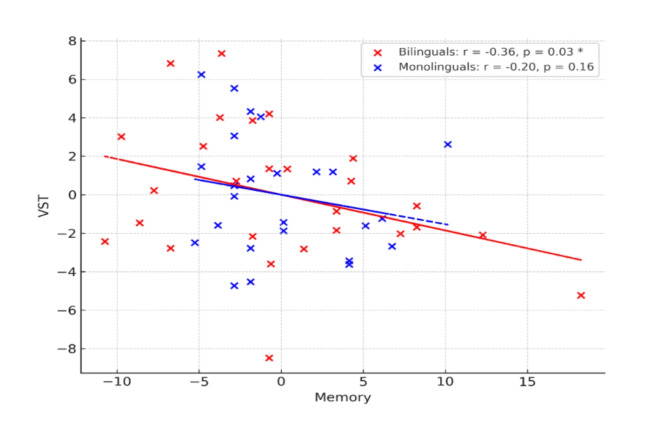
Scatter plot illustrating the relationship between performance on the episodic memory and inhibitory control tests, with residuals for both measures adjusted for sex. Red crosses indicate bilinguals and blue ones indicate monolinguals. Asterix denotes significant relationship (1-tailed partial correlation).

Across both groups combined, we found a positive relationship between memory performance and inhibitory control ((*r*(49) = -0.32, *p* = .01; correlation is negative because lower scores indicate better inhibitory control). We further tested for such relations in the groups separately and found a significant relationship in the bilingual group only (*r*(24) = -0.36, *p* = .03). In other words, in bilinguals, better performance on WWW test of episodic memory correlated positively with better inhibitory control. There was no association for the monolinguals (*r*(22) = -0.20, *p* = .17), although the relationship was in the same direction in this group. We further tested to see if the correlations in the two groups differed statistically, which they didn’t (z-score = -0.59, *p* = .56). Figure 1 shows scatter plots illustrating the relationship.

### Brain imaging results

#### Group differences

##### Group differences in volume of ROIs

**Fig. 2:**
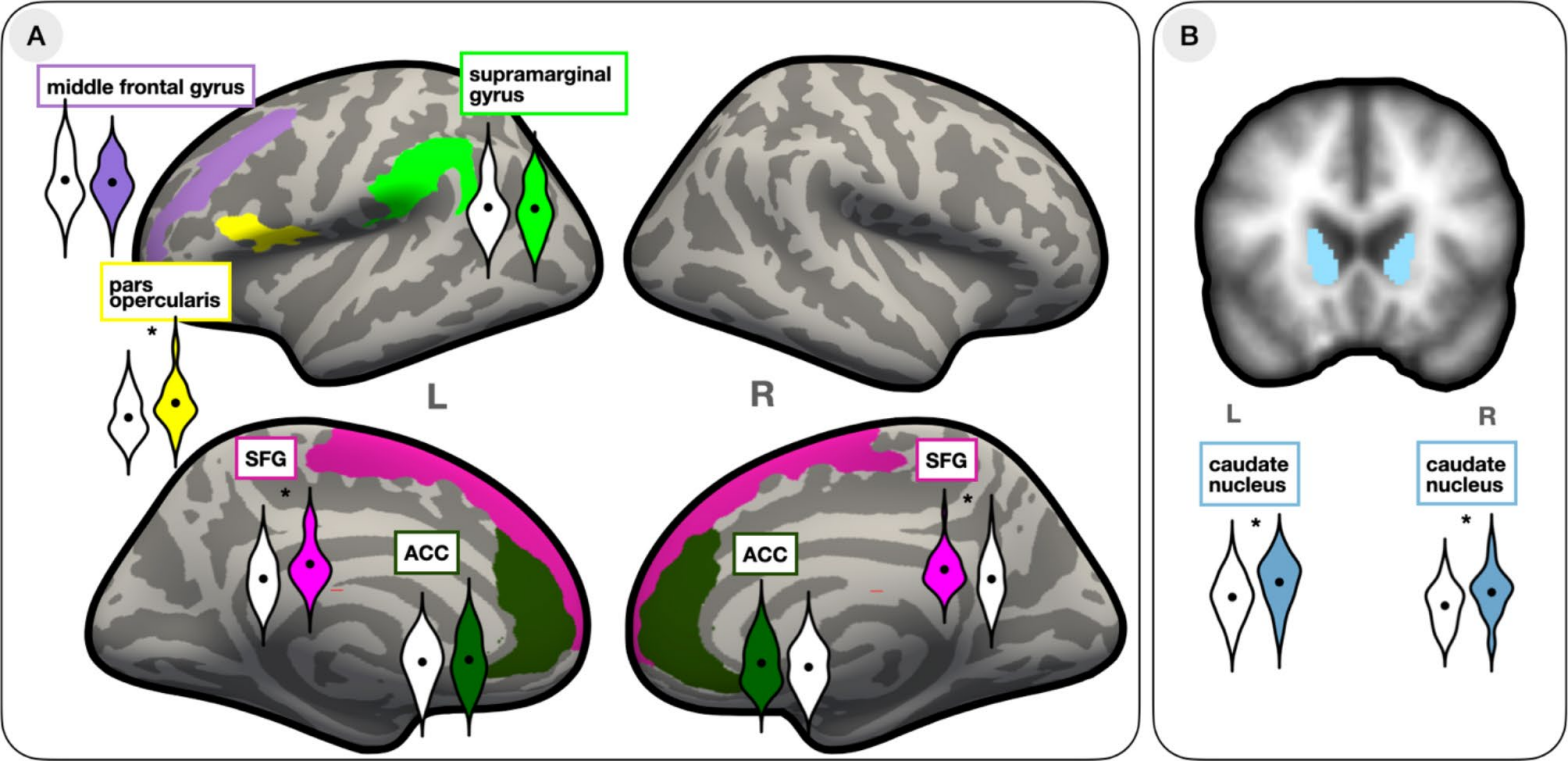
Results of the group differences analyses in the volume of ROIs between monolinguals (white violins) and bilinguals (coloured violins). The violin plot outlines illustrate the density of the data; black circles represent group means. Significant results are marked with (*). **(A)** Results of the investigated cortical ROIs (left middle frontal gyrus, left pars opercularis, left supramarginal gyrus, left and right SFG, left and right anterior cingulate cortex; coloured labels show corresponding Freesurfer ROIs). **(B)** Group differences in the volume of the caudate nucleus.

Results are summarized in Fig. 2; Table 2. As can be seen from Table 2, there were larger grey matter volumes in the bilinguals in the following regions: the left pars opercularis of the inferior frontal gyrus, and in bilateral SFG and caudate nuclei (Fig. 2).

**Table 2:** Group differences: MANCOVA examining group differences in grey matter volume (mean (SD)) of selected ROIs (covariates: sex and whole hemispheric volume, *df* = 1,48 for all tests).

ROIs	*F*-value	*p*-value	*GM volume – mean (SD)*	
			Monolinguals	Bilinguals
Left pars opercularis	8.70**	0.004	3179 (426)	3504 (504)
Left supramarginal gyrus	0.07	0.79	6704 (957)	6654 (942)
Left middle frontal gyrus	0.27	0.60	10,208 (1778)	10,042 (1448)
Left caudate nucleus	7.22**	0.01	3604 (340)	3834 (360)
Right caudate nucleus	6.30**	0.02	3810 (321)	4026 (383)
Left SFG	12.02**	0.001	15,444 (1916)	16,768 (2031)
Right SFG	6.31**	0.01	14,676 (1822)	15,412 (1395)
Left ACC	0.36	0.55	4359 (604)	4421 (693)
Right ACC	1.01	0.32	5660 (798)	5805 (837)

** indicates significant difference (*p* < .05).

##### Group differences in volume of hippocampus, anterior and posterior hippocampus, and parasubiculum

It can be seen from Table 3 that there were larger grey matter volumes in the bilinguals on the left (*F*(1,48) = 4.16, *p* = .02, η*p*^2^ = 0.08) and right parasubiculum (*F*(1,48) = 4.67, *p* = .02, η*p*^2^ = 0.09). Volumes of the left anterior hippocampus (*F*(1,48) = 2.88, *p* = .05, η*p*^2^ = 0.06), and the right anterior hippocampus (*F*(1,48) = 2.00, *p* = .08, η*p*^2^ = 0.04) did not reach statistical significance. Note, however, that the effect sizes are progressively bigger, from η*p*^2^ = 0.0002 and η*p*^2^ = 0.002, and being non-significant for the largest ROI (bilateral hippocampus), to η*p*^2^ = 0.06 and being significant in the left anterior hippocampus (and η*p*^2^ = 0.04 and *p* = .08 for the right), and finally with the highest effect sizes and lowest *p*-values for the parasubiculum, bilaterally. The group difference was not significant in the posterior hippocampi.

**Table 3:** Group differences: MANCOVA examining group differences in grey matter volume of the bilateral whole hippocampus, anterior and posterior hippocampi and parasubiculum (covariates: sex and whole hemispheric volume, *df* = 1,48 for all tests).

ROIs	*F*-value	*p*-value
Left hippocampus	0.01	0.46
Left anterior hippocampus	2.88	0.05
Left parasubiculum	4.16	0.02*
Left posterior hippocampus	0.76	0.39
Right hippocampus	0.09	0.39
Right anterior hippocampus	2.00	0.08
Right parasubiculum	4.67	0.02*
Right posterior hippocampus	0.41	0.53

* indicates significant difference (*p* < .05).

#### Brain–behaviour relationships

Table 4 illustrates the result of partial correlations, controlling for sex and cortical volume, to test for relationships between episodic memory performance and the volume of the different progressively more selective ROIs (as above), across the groups combined. We additionally examined the groups separately, to test for possible group differences in the strength of the relationship between brain structure and behaviour (see Table 5).

**Table 4:** Partial correlations across both groups between performance on the episodic memory test and grey matter volumes of relatively more to relatively less inclusive hippocampal/parahippocampal combinations of regions, controlling for sex and whole brain cortex volume.

ROIs	*R*-value	*p*-value
Left hippocampus	0.40	0.002*
Left anterior hippocampus	0.21	0.07
Left parasubiculum	0.16	0.14
Left posterior hippocampus	0.32	0.01*
Right hippocampus	0.31	0.02*
Right anterior hippocampus	0.05	0.35
Right parasubiculum	0.25	0.04*
Right posterior hippocampus	0.32	0.01*

* indicates a significant difference (*p* < .05).

**Table 5:** Partial correlations in the bilingual and monolingual groups separately between performance on the episodic memory test and grey matter volumes of relatively more to relatively less inclusive hippocampal / parahippocampal of regions, controlling for sex and whole brain cortex volume.

	Bilinguals		Monolinguals	
*ROIs*	*R-value*	*p-value*	*R-value*	*p-value*
Left hippocampus	0.49	.006*	0.31	.07
Left anterior hippocampus	0.24	.13	0.02	.45
Left parasubiculum	0.23	.13	-0.18	.20
Left posterior hippocampus	0.47	.003*	0.34	.06
Right hippocampus	0.41	.02*	0.20	.19
Right anterior hippocampus	-0.02	.45	-0.08	.35
Right parasubiculum	0.33	.05	-0.23	.15
Right posterior hippocampus	0.56	.002*	0.23	.15

* indicates a significant difference (*p* < .05).

In order to further explore the question of the effect of bilingualism and hippocampal volumes on memory, we fitted two linear regression models predicting memory performance from the volume of the left hippocampus and the volume of the left anterior hippocampus (with sex and hemispheric volume as covariates). We observed that both variables (bilingualism and hippocampal volumes) remained significant predictors of the memory performance, pointing to the interpretation that bilingualism is associated with better memory scores above and beyond the volume of the hippocampus. All variables had a medium effect size, but in the model predicting memory scores from the left anterior hippocampal volume, bilingualism was a better predictor (fp2 = 0.28 versus fp2 = 0.20).

#### Whole brain structural covariance analyses

**Fig. 3:**
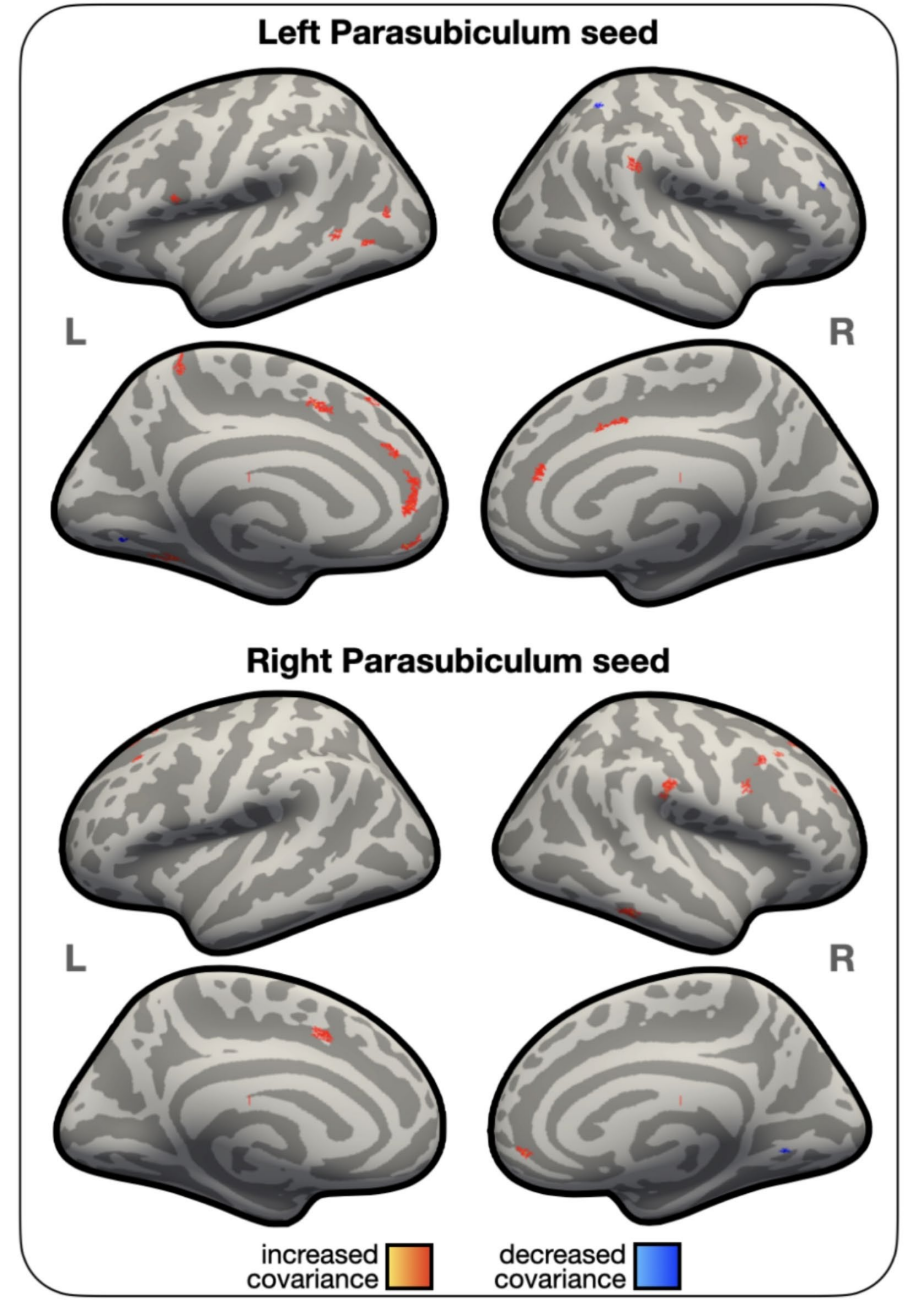
Results of the vertex-wise covariance with volume of the left (top panel) and right (bottom panel) parasubiculum across all participants.

The structural covariance analyses revealed positive covariance between the left parasubiculum volume and that of bilateral rostral anterior cingulate cortex, left SFG and medial orbitofrontal gyri, left pars opercularis, left fusiform and lateral occipital gyri, left planum temporale and MTG, left precuneus, and right supramarginal and posterior cingulate gyri. The right parasubiculum volume covaried positively with that of bilateral SFG, medial orbitofrontal gyri, caudal MFG, left fusiform gyrus, and with right supramarginal gyrus, rostral MFG, MTG, and precentral gyrus. Negative structural covariance was found with much fewer regions, including the left lingual gyrus and right superior parietal and rostral MFG for the left parasubiculum, and the right lingual and inferior temporal gyri for the right parasubiculum (Fig. 3 and Supplementary Table 1). Note that because this analysis was done in vertex space, subcortical regions were not included.

## Discussion

Our behavioural results showed that Persian-Kurdish bilinguals outperformed monolinguals on the nonverbal WWW test of episodic memory, extending previous such findings in older adults^5^ and children^4^ to young adults. In line with the explanation proposed in the latter study, the bilingual episodic memory advantage observed in our study could arise from a greater need for bilinguals to integrate and/or organize information in their two languages, which could then lead to cognitive advantages in other domains, including memory^4^. Alternatively and non-exclusively, the advantage could be explained by the fact that according to the bilingual dual-coding theory, bilinguals form distinct verbal codes for each of their languages, and this could boost their memory system by allowing for deeper encoding and by providing two retrieval paths^55^. Thus, our result endorses the idea that bilingualism can result in benefits for episodic memory.

We did not find evidence, however, for better performance in the bilinguals on the VST of inhibitory control, despite the fact that we did find group brain structural differences in regions known to be involved in executive control (see below). Several previous studies also found no bilingual advantage on the Stroop or related tasks^3^, while others did find better inhibitory control in bilinguals^1^. It could be that the bilingual executive control advantage on executive control is either weak (or even non-existent)^3^, or that bilingual advantages on executive and other outcome measures depend on the age range tested, on the duration and extent of bilingualism, on the typological distance between languages spoken, and/or other factors^2,56^. Our participants were simultaneous, proficient bilinguals who used their two languages daily (see Table 1), and it is thus more likely that they have less conflict between their languages^57^. It is thus possible that the same test would have shown group differences in late and/or non-proficient bilinguals, or in bilinguals who speak pairs of languages that are more (or less, due to greater interference) typologically distant than Persian and Kurdish. Our null finding could also be due to the fact that our participants were healthy young adults, who performed at ceiling. Indeed, better executive control in bilinguals has most often been detected either in children or in older adults, and several studies have proposed that, unlike older adults and children, young adults have high executive control abilities already^58^. Thus, the VST task might not have been sufficiently challenging or not sensitive enough, and in light of the observed group differences in some executive control brain regions, it is possible that a more difficult task would reveal an inhibitory control advantage in our bilingual participants. Finally, due to the early and simultaneous nature of bilingualism in our participants, it is possible that bilingualism-related executive control advantages that are hypothesized to have been present earlier in life in our participants have progressively faded, leading to equal inhibitory control skills as found in monolinguals.

Interestingly, we found a positive correlation between episodic memory performance and inhibitory control. This relationship was significant only in the bilingual group, although the difference in the strength of the correlation didn’t differ between groups, making it difficult to rule out it’s absence in monolingual individuals. Our findings extend those of a study showing a relationship between episodic memory and cognitive control/attention (assessed via the Simon task) in older bilingual (but not monolingual) adults^5^, where it was suggested that better executive control arising from the control requirements of bilingualism could lead to more controlled encoding and retrieval in episodic memory. Conversely and non-exclusively, it could be that the greater language and vocabulary learning in bilinguals leads to beneficial cascade effects on higher-level executive control. Indeed, as recently estimated, the majority of information stored by proficient language users relates to lexical semantics^59^, and, by definition, multilinguals, have to accumulate such lexical knowledge across more than one language.

Our brain structural imaging results revealed larger grey matter volumes in bilinguals in the left pars opercularis of the inferior frontal gyrus (posterior part of ‘Broca’s area’), known to be involved in phonological, semantic and syntactic processing, with longitudinal studies having shown evidence for structural plasticity of this region following second language learning^60^. A larger left pars opercularis in the bilinguals may reflect the greater need to regularly use and master different levels of linguistic information in bilinguals compared to monolinguals. We also found larger gray matter volumes in the bilinguals in the SFG (with the whole brain analysis showing extension to the supplementary motor area (SMA)), and in the caudate nuclei bilaterally. The bilateral SFG is a well-established component of the executive and cognitive control network, and the right SFG has been implicated in inhibitory control^24^. Although the caudate nucleus is involved in many aspects of functioning including learning and reward, it is also known to be an important part of cognitive and also language control networks in the brain^53^. Anatomical studies have also shown larger volumes of the right or of bilateral caudate nuclei in bi- and multilinguals compared to monolinguals^12,61^. The larger volumes of these regions in our bilingual participants may reflect executive / language control benefits in our bilinguals that were not detected by the Stroop task. Alternatively, it is possible that bilingualism-related executive control advantages that are hypothesized to have been present earlier in life have progressively faded in our early, simultaneous bilinguals, but that the brain structural differences that may have arisen due to the demands earlier in life related to the challenge of acquiring and managing two languages have remained detectable in our bilingual participants.

We also found larger grey matter volumes of the parasubiculum bilaterally, with smaller effects (which were marginally above the customary statistical threshold level of 0.05) in bilateral anterior hippocampi (left: η*p*^2^ = 0.06; right: η*p*^2^ = 0.04). Previous studies on hippocampus anatomy in bilinguals have looked at the hippocampus as a whole ^17,18^, despite the fact that there are numerous hippocampal subregions. Further, there’s evidence that the anterior and posterior hippocampi may have differential roles in language- vs. episodic memory-related functions, respectively, and that the parasubiculum (located in the anterior hippocampus) is involved in episodic memory when scene-based information is involved^30–32,34^. Significantly larger volumes of bilateral parasubiculum and a tendency towards a larger left anterior hippocampi in our bilinguals suggest that the greater memory requirements of learning and using two languages result in neuroplastic changes in these regions. Parasubiculum and anterior hippocampus are known to be involved in language knowledge and in (scene-based) episodic memory, and to be functionally and anatomically connected to anterior and lateral temporal and inferior frontal lobes^30,62^, important components of the brains language and semantic memory network. Our findings are also consistent with those of brain structural imaging results having shown a relationship between the volume of the anterior hippocampus and verbal memory, and of posterior subregion volumes and visual memory^63^. Although our study is cross-sectional, previous longitudinal studies have shown evidence for structural plasticity in the hippocampus following language learning^17,18^, and our work helps to pinpoint the location within the hippocampus that may underlie such learning-related changes. Our participants were non-elective bilinguals (i.e. they became bilingual due to environmental requirements rather than to high language learning aptitude), lending support to the interpretation that the observed group differences were due to experience (see^64^, for a similar interpretation proposed for their structural group differences in the auditory cortex).

Individual differences across all participants in episodic memory performance correlated with the volume of bilateral hippocampus. Exploration of the regional differences contributing to this correlation located the relationship to the bilateral posterior hippocampi, in line with evidence for involvement of this region in episodic memory. The relationship was also significant at the group level in the right parasubiculum, shown by previous work to be involved bilaterally in episodic memory retrieval from autobiographical memory^38^, and in the mental construction of internal scenes during episodic memory recall^39^. This is consistent with the heavy spatial nature of our episodic memory task, in which participants were required to retrieve information not only about what object was hidden and when but also about where it was hidden, in a virtual scene. Analysis of correlations within each group separately only showed significant results in the bilinguals (again in bilateral hippocampi, localised to bilateral posterior hippocampi, this time with a strong trend (*p* = .05) in the right parasubiculum), suggesting again that relationships between episodic memory and hippocampal anatomy may be modulated by bilingualism. Voits and colleagues^6^ had found a negative relationship between episodic memory performance and hippocampal volume, in older adults. The direction of our findings is positive and localised more precisely to the posterior hippocampi and the right parasubiculum. The inconsistency in the results could be because of the different age groups tested in the two studies. Further, it is also possible that the result in older adults, if probed for subregional localisation, is positive in posterior hippocampus but negative in other regions, and that in older adults the negative relationship overrides the possibly more localised positive one. In monolinguals there were no significant relationships, but only a strong trends in the left hippocampus (*p* = .07), localised to the left posterior hippocampus (*p* = .06). Episodic memory skill is likely influenced by many other aspects of experience beyond bilingualism, such as the amount of education, cognitive and physical activity levels, musical experience, previously shown to be beneficial to cognitive and neural reserve. The trends we observe in the monolinguals are likely to be driven by individual differences in such other experience-related factors, and such other experiences are likely to also contribute to the relationships in the bilinguals, beyond the bilingual experience. Our interpretation is supported by follow-up analyses which showed that both bilingualism and hippocampal volumes significantly predicted memory performance – but with a stronger contribution of bilingualism, suggesting that bilingualism is associated with better memory scores above and beyond the volume of the hippocampus.

Finally, we found evidence for positive structural covariance between the bilateral parasubiculum and regions including important components of the executive control network, including the bilateral rostral anterior cingulate cortex, the left SFG and medial orbitofrontal gyri and the left pars opercularis for the left parasubiculum, and including the bilateral SFG, medial orbitofrontal gyri and caudal middle frontal gyri for the right parasubiculum. Interestingly, there was also positive covariance between the left parasubiculum and areas known to be involved in language, such as the left pars opercularis, planum temporale, MTG and fusiform gyrus, and between the right parasubiculum and the left fusiform gyrus. We hypothesize that the higher memory requirements of bilingualism, which we propose lead to plastic change in the bilateral parasubiculum, in turn resulted in cascade effects in the executive control network. Here again, non-exclusively, the converse may hold: it could be that extensive practice controlling two languages may result in structural plasticity in executive control regions, which due to posited functional and structural connectivity with the anterior hippocampus can in turn result in improved (episodic) memory in bilinguals. Our results suggest that bilingual experience is one important experiential factor that leads to reciprocal boosting of memory and of executive brain networks, and that increases in behavioural skills are associated with this neural plasticity, at least in episodic memory in our participants. Increased covariance between the parasubiculum and brain regions known to be important for language processing further supports the anatomical links between this anterior hippocampus subregion and the language network in the brain, in line with known evidence for anterior hippocampus being linked to language knowledge. Our group differences and structural covariance findings are consistent with those of a recent cross-sectional study in a very large sample of mono- and bilingual individuals spanning the ages of 3–21, which showed that compared to monolinguals, bilinguals showed less developmental loss, starting during late childhood and adolescence, in regions including the pars opercularis and SFG^9^. Slower age-related decline has also been shown in the hippocampal volumes of bilingual compared to monolingual adults^6,19^. Our study was not longitudinal, so future longitudinal work is needed to show the dynamics of these bilingualism related differences in the hippocampus (subregions) compared to in language, memory and executive control regions, to determine which causes which, or whether the observed changes/differences happen in parallel.

We also found negative structural covariance between the left parasubiculum seed and the left lingual gyrus, right superior parietal region and rostral middle frontal region, and between the right parasubiculum seed and the right inferior temporal and lingual gyri. These regions are known to be functionally implicated in visual functions (e.g. object recognition), in aspects of spatial attention and awareness, and in face processing. It’s possible that these regions show negative covariance with parasubiculum volumes due to the known complementary (and possibly competitive) specialisation for visual aspects of episodic memory (supported by regions such as the right inferior temporal cortex) versus verbal, conceptual ones.

We found that group differences versus correlations with episodic memory performance are different in hippocampal subregions, with the former being localised to the bilateral parasubiculum and with trends in the bilateral anterior hippocampus, versus the latter being localised to the bilateral posterior hippocampus and the right parasubiculum. These partial dissociations are consistent with the predicted preferential role of the anterior hippocampus in language knowledge, and the finer localisation to the bilateral parasubiculum highlights the possible further role of this region in aspects of language learning. Future studies are needed to further understand what the functional role of this region is in language learning, and also to replicate this finding in larger samples. Finally, it remains to be seen whether this finding would also be observed in later and/or less proficient bilinguals, or if it is specific to simultaneous and proficient bilingualism.

The findings could be useful for educational policymakers to promote the incorporation of bilingual education programs, particularly in regions in Iran with ethnolinguistic diversity. Introducing Kurdish alongside Persian for primary school children offers numerous cognitive and sociocultural benefits. Cognitively, our work supports the idea that early bilingualism strengthens brain regions responsible for executive control and memory, possibly promoting other cognitive advantages (not tested here). On a socio-cultural level, it fosters a sense of pride and identity among Kurdish-speaking children, promoting cultural inclusivity and reducing the marginalization of minority groups. It encourages respect for linguistic diversity and empowers children to maintain their heritage. This dual language exposure strengthens communication between generations and communities. By supporting bilingual education, schools can help children thrive academically while fostering a deeper understanding of their cultural roots and promoting social cohesion. Parents can also benefit by exposing children to both Kurdish and Persian at home which in turn plays a crucial role in recalling personal experiences and learning new information since a stronger episodic memory can facilitate better retention of daily events and lessons. Also, children with superior episodic memory may find it easier to link new concepts to past experiences, enriching their understanding of complex topics.

### Limitations and future directions

The current, cross-sectional study involved looking at behavioural performance and brain structural measures of volume and of structural covariance. As such, the conclusions that we can draw about the cognitive and neural mechanisms underlying the posited interacting memory and executive control bilingual benefits and their brain structural underpinnings are limited. Structural covariance analyses, which we report here, are informative regarding the structural differences that go hand in hand with those of a seed ROI across a sample of individuals, but do not directly speak to underlying differences in brain functional or structural connectivity. Previous diffusion tensor imaging studies have shown the critical role of white matter tracts connecting the hippocampus to superior frontal regions in supporting episodic memory^65,66^, but further work is needed to examine such connectivity in relation to individual differences in bilingual experience and executive skill. Thus, although our study provides the first empirical work tackling the question of how two cognitive domains could be related to one another and to brain structural differences, future brain functional and structural connectivity (i.e. diffusion weighted imaging) studies are needed to further probe our new account of how bilingual language experience may impact memory and executive cognitive abilities and their underlying neural underpinnings. Also, as in most studies in the field, we used one behavioural test per cognitive domain, and therefore replication using other tests tapping into these domains of processing is necessary to strengthen the conclusions that can be drawn.

Generalisability to other bilingual groups who speak other language combinations remains to be tested in future studies. Our study, like many in the field of cognitive neuroscience, was performed in university students, and as such, generalisability to bilinguals from different socio-cultural backgrounds is an open question. It is possible for example that participants from a lower SES would show stronger effects of bilingualism on (episodic) memory and in the anatomy of hippocampal (sub)regions, since the learning and use of a second language could play a relatively bigger role in modulating cognitive reserve and (episodic) memory in these individuals (due to lower education levels). Generalization to other pairs of languages also remains to be explored in future work. Persian and Kurdish are sister languages, and it is possible that bilingual experience in pairs of languages that differ more typologically would lead to greater memory and neural benefits. Finally, the study was performed within a sociolinguistic environment in which both languages are spoken on a day-to-day basis, likely necessitating regular switching between the languages, placing a higher cognitive load on working memory and episodic memory processes, and regular monitoring and management of two separate language systems. Generalisation to bilinguals who don’t use both of their languages on a day-to-day basis remains to be tested.

Our study was cross-sectional in its design, which on the one hand allows to test differences related to lifelong bilingual experience, but which on the other hand is limited in terms of the interpretation of the results regarding causality. Indeed, although it is tempting to believe that it is bilingual experience that leads to changes in episodic memory and to differences in the volume of hippocampal subregions, it could alternatively be that people with better episodic memory or with pre-existing bigger hippocampal subfield volumes are more likely to learn a second language. Our participants were, however, non-elective bilinguals who became bilingual because of the requirements of their socio-cultural environment, making the latter interpretations less likely. Longitudinal studies on language learning showing effects on the anatomy of the hippocampus and of other brain regions already exist^13,17,18^, and an impressive longitudinal behavioural study over the span of more than 50 years showed advantages of bilingualism for general intelligence and for reading - beyond what would have been predicted from baseline (i.e. time point 1) cognitive abilities^67^. Although such longer term longitudinal studies on bilingual benefits including brain imaging are very challenging to do over many years of learning, such efforts would provide very important insights into the developmental and lifespan trajectory of bilingual benefits, and allow to see how memory and executive control benefits change dynamically and in interaction with one another, and how these play out in terms of changes in brain function, structure, and functional and structural connectivity.

## Conclusions

Overall, our findings confirm that experience from birth with two non-European languages – ones that differ in terms of their typologies at multiple linguistic levels – fosters possible brain structural adaptations (i.e., plasticity) in brain regions involved in linguistic processing but also in ones involved in memory and in executive processing, even in young simultaneous, proficient bilingual adults. It is possible that non-simultaneous bilingualism would be related to different (possibly higher) such benefits, and likely that these effects would be more pronounced in older adults. This work brings forth a novel framework within which to integrate the literature on the executive control and memory benefits of bilingualism, providing a different explanation for the bilingual advantage that has been provided thus far. It also has important implications for the protective role of bilingualism on cognition and on the brain, not only in aging but possibly also in other contexts in which performance is challenged, for example in the context of developmental (learning) and other disorders that affect cognitive function and its neural underpinnings.

## Electronic supplementary material

Below is the link to the electronic supplementary material.

Supplementary Material 1

Supplementary Material 2

## Data Availability

The datasets used and/or analysed during the current study are available from the corresponding author upon reasonable request.
