# Supplementary material for: Neuroanatomy, episodic memory and inhibitory control of Persian-Kurdish simultaneous bilinguals
Source: Sci Rep. 2024 Nov 25;14:29151. doi: 10.1038/s41598-024-79955-2 (PMC11589158; doi:10.1038/s41598-024-79955-2)
Supplement: Supplementary file 1 — Supplementary Material 1 [file 41598_2024_79955_MOESM1_ESM.pdf]

Supplementary Table S1.

Clusters of significant vertex-wise covariance with left and right parasubiculum. Both positive and negative covariance are reported

Max	*Size (mm²)*	*MNIX*	*MN1*	*MNIZ*	*WghtVtx*	*Region*
				**Seed: left parasubiculum**		
**Left hemisphere**						
Positive structural connectivity						
4.444	80.63	-10.2	42.7	4.2	305.00	Rostral Anterior Cingulate
5.256	68.15	-33.2	-50.7	-10.0	274.00	Fusiform
5.165	35.64	-10.2	6.9	43.3	175.12	Superior Frontal
3.972	37.09	-14.5	-47.1	60.4	174.10	Precuneus
3.655	28.91	-48.0	7.4	10.1	107.58	Pars Opercularis
4.428	33.55	-10.9	42.1	-11.3	103.26	Medial Orbitofrontal
3.639	29.73	-56.0	-54.0	3.4	93.48	Banks of Superior Temporal
3.746	23.40	-50.8	-61.7	-1.8	85.86	Middle Temporal
3.713	28.32	-12.4	36.6	23.1	82.06	Superior Frontal
3.229	20.28	-45.3	-70.4	8.8	68.50	Lateral Occipital
2.934	18.07	-7.6	27.0	48.8	57.60	Superior Frontal
4.592	16.37	-61.4	-30.1	-16.9	52.18	Middle Temporal
4.821	16.54	-5.8	29.9	-19.3	48.00	Medial Orbitofrontal
3.280	20.34	-19.7	-99.5	2.7	40.74	Lateral Occipital
Decreased structural connectivity						
-2.791	23.30	-15.0	-69.6	-7.4	-49.17	Lingual
**Right hemisphere**						
Positive structural connectivity						
4.650	50.02	61.2	-39.7	32.7	218.81	Supramarginal
4.968	49.06	45.4	-0.3	39.3	144.64	Precentral

3.820	31.01	8.6	2.1	39.3	140.05	Posterior Cingulate
3.778	29.34	11.7	36.1	14.3	112.57	Rostral Anterior Cingulate
Negative structural connectivity						
-3.102	22.18	31.6	-50.5	53.1	-75.88	Superior Parietal
-3.521	18.05	40.4	35.8	21.3	-58.09	Rostral Middle Frontal
				**Seed: right parasubiculum**		
**Left hemisphere**						
Positive structural connectivity						
5.530	38.54	-40.8	-31.8	-21.8	163.15	Fusiform
3.501	32.87	-11.0	10.2	40.5	168.43	Superior Frontal
4.793	30.02	-12.6	24.3	54.3	114.03	Superior Frontal
4.214	24.06	-19.9	32.3	49.7	81.26	Superior Frontal
3.512	20.12	-32.3	22.8	42.9	64.41	Caudal Middle Frontal
3.544	20.04	-19.6	15.5	57.4	61.69	Superior Frontal
3.560	19.96	-19.9	31.8	46.3	54.21	Superior Frontal
3.638	17.90	-38.3	-49.1	-20.2	71.10	Fusiform
3.646	17.03	-21.3	22.1	56.1	45.65	Superior Frontal
2.550	16.58	-6.4	57.8	-11.1	28.66	Medial Orbitofrontal
3.011	16.08	-39.1	18.2	45.6	47.29	Caudal Middle Frontal
**Right hemisphere**						
Positive structural connectivity						
5.040	59.46	57.2	-26.2	27.5	334.82	Supramarginal
6.095	60.78	21.8	27.5	45.7	181.47	Superior Frontal
4.561	49.36	54.7	-37.2	-15.3	158.10	Middle Temporal
3.972	26.10	44.8	2.9	29.7	99.66	Precentral

3.070	34.50	37.7	11.2	39.6	96.52	Caudal Middle Frontal
5.083	21.92	35.0	21.6	47.7	84.24	Caudal Middle Frontal
3.306	27.08	25.6	43.6	29.1	77.92	Rostral Middle Frontal
4.287	17.72	12.0	41.7	-6.8	76.42	Medial Orbitofrontal
4.625	20.52	19.7	53.4	22.3	60.58	Rostral Middle Frontal
Negative structural connectivity						
-2.729	17.80	39.9	-5.5	-39.2	-51.34	Inferior Temporal
-3.914	17.67	9.9	-59.8	-0.8	-59.79	Lingual
