# Supplementary material for: Neuroanatomy, episodic memory and inhibitory control of Persian-Kurdish simultaneous bilinguals
Source: Sci Rep. 2024 Nov 25;14:29151. doi: 10.1038/s41598-024-79955-2 (PMC11589158; doi:10.1038/s41598-024-79955-2)
Supplement: Supplementary file 2 — Supplementary Material 2 [file 41598_2024_79955_MOESM2_ESM.docx]

**Neuroanatomy, episodic memory and inhibitory control of Persian-Kurdish simultaneous bilinguals**

Samira Golshani^1^, Olga Kepinska^3,4^, Hamid Gholami#*^1^, Narly Golestani#^3,4,5^

^1^ Department of Humanities, Kermanshah Branch, Islamic Azad University, Kermanshah, Iran.

^3^ Brain and Language Lab, Cognitive Science Hub, University of Vienna, Vienna, Austria.

^4^ Department of Behavioral and Cognitive Biology, Faculty of Life Sciences, University of Vienna, Vienna, Austria.

^5^ Brain and Language Lab, Department of Psychology, Faculty of Psychology and Educational Sciences, University of Geneva, Switzerland

*Corresponding email: [hamid.gholami@iau.ac.ir](mailto:hamid.gholami@iau.ac.ir)

# Joint senior authors contributed equally to this work.

**Supplementary Table S1.**

Clusters of significant vertex-wise covariance with left and right parasubiculum. Both positive and negative covariance are reported

| ***Max*** | ***Size (mm^2^)*** | ***MNIX*** | ***MNIY*** |  | ***MNIZ*** | ***WghtVtx*** | ***Region*** |
| --- | --- | --- | --- | --- | --- | --- | --- |
| **Seed: left parasubiculum** | | | | | | | |
| **Left hemisphere** | | | | | | | |
| Positive structural covariance | | | | | | | |
| 4.444 | 80.63 | -10.2 | 42.7 |  | 4.2 | 305.00 | Rostral Anterior Cingulate |
| 5.256 | 68.15 | -33.2 | -50.7 |  | -10.0 | 274.00 | Fusiform |
| 5.165 | 35.64 | -10.2 | 6.9 |  | 43.3 | 175.12 | Superior Frontal |
| 3.972 | 37.09 | -14.5 | -47.1 |  | 60.4 | 174.10 | Precuneus |
| 3.655 | 28.91 | -48.0 | 7.4 |  | 10.1 | 107.58 | Pars Opercularis |
| 4.428 | 33.55 | -10.9 | 42.1 |  | -11.3 | 103.26 | Medial Orbitofrontal |
| 3.639 | 29.73 | -56.0 | -54.0 |  | 3.4 | 93.48 | Banks of Superior Temporal Sulcus |
| 3.746 | 23.40 | -50.8 | -61.7 |  | -1.8 | 85.86 | Middle Temporal |
| 3.713 | 28.32 | -12.4 | 36.6 |  | 23.1 | 82.06 | Superior Frontal |
| 3.229 | 20.28 | -45.3 | -70.4 |  | 8.8 | 68.50 | Lateral Occipital |
| 2.934 | 18.07 | -7.6 | 27.0 |  | 48.8 | 57.60 | Superior Frontal |
| 3.280 | 20.34 | -19.7 | -99.5 |  | 2.7 | 40.74 | Lateral Occipital |
| Negative structural covariance | | | | | | | |
| -2.791 | 23.30 | -15.0 | -69.6 |  | -7.4 | -49.17 | Lingual |
| **Right hemisphere** | | | | | | | |
| Positive structural covariance | | | | | | | |
| 4.650 | 50.02 | 61.2 | -39.7 |  | 32.7 | 218.81 | Supramarginal |
| 4.968 | 49.06 | 45.4 | -0.3 |  | 39.3 | 144.64 | Precentral |
| 3.820 | 31.01 | 8.6 | 2.1 |  | 39.3 | 140.05 | Posterior Cingulate |
| 3.778 | 29.34 | 11.7 | 36.1 |  | 14.3 | 112.57 | Rostral Anterior Cingulate |
| Negative structural covariance | | | | | | | |
| -3.102 | 22.18 | 31.6 | -50.5 |  | 53.1 | -75.88 | Superior Parietal |
| -3.521 | 18.05 | 40.4 | 35.8 |  | 21.3 | -58.09 | Rostral Middle Frontal |
| **Seed: right parasubiculum** | | | | | | | |
| **Left hemisphere** | | | | | | | |
| Positive structural covariance | | | | | | | |
| 5.530 | 38.54 | -40.8 | -31.8 |  | -21.8 | 163.15 | Fusiform |
| 3.501 | 32.87 | -11.0 | 10.2 |  | 40.5 | 168.43 | Superior Frontal |
| 4.793 | 30.02 | -12.6 | 24.3 |  | 54.3 | 114.03 | Superior Frontal |
| 4.214 | 24.06 | -19.9 | 32.3 |  | 49.7 | 81.26 | Superior Frontal |
| 3.512 | 20.12 | -32.3 | 22.8 |  | 42.9 | 64.41 | Caudal Middle Frontal |
| 3.544 | 20.04 | -19.6 | 15.5 |  | 57.4 | 61.69 | Superior Frontal |
| 3.560 | 19.96 | -19.9 | 31.8 |  | 46.3 | 54.21 | Superior Frontal |
| 3.638 | 17.90 | -38.3 | -49.1 |  | -20.2 | 71.10 | Fusiform |
| 3.646 | 17.03 | -21.3 | 22.1 |  | 56.1 | 45.65 | Superior Frontal |
| **Right hemisphere** | | | | | | | |
| Positive structural covariance | | | | | | | |
| 5.040 | 59.46 | 57.2 | -26.2 |  | 27.5 | 334.82 | Supramarginal |
| 6.095 | 60.78 | 21.8 | 27.5 |  | 45.7 | 181.47 | Superior Frontal |
| 4.561 | 49.36 | 54.7 | -37.2 |  | -15.3 | 158.10 | Middle Temporal |
| 3.972 | 26.10 | 44.8 | 2.9 |  | 29.7 | 99.66 | Precentral |
| 3.070 | 34.50 | 37.7 | 11.2 |  | 39.6 | 96.52 | Caudal Middle Frontal |
| 5.083 | 21.92 | 35.0 | 21.6 |  | 47.7 | 84.24 | Caudal Middle Frontal |
| 3.306 | 27.08 | 25.6 | 43.6 |  | 29.1 | 77.92 | Rostral Middle Frontal |
| 4.287 | 17.72 | 12.0 | 41.7 |  | -6.8 | 76.42 | Medial Orbitofrontal |
| 4.625 | 20.52 | 19.7 | 53.4 |  | 22.3 | 60.58 | Rostral Middle Frontal |
| Negative structural covariance | | | | | | | |
| -2.729 | 17.80 | 39.9 | -5.5 |  | -39.2 | -51.34 | Inferior Temporal |
| -3.914 | 17.67 | 9.9 | -59.8 |  | -0.8 | -59.79 | Lingual |
